# Time-series modelling and forecasting of hand, foot and mouth disease cases in China from 2008 to 2018

**DOI:** 10.1017/S095026881800362X

**Published:** 2019-01-31

**Authors:** C. W. Tian, H. Wang, X. M. Luo

**Affiliations:** Department of Infectious Diseases, Kunshan Centers for Disease Control and Prevention, Jiangsu Province, China

**Keywords:** Hand, monthly incidence, foot and mouth disease, seasonal autoregressive-integrated moving average model

## Abstract

Seasonal autoregressive-integrated moving average (SARIMA) has been widely used to model and forecast incidence of infectious diseases in time-series analysis. This study aimed to model and forecast monthly cases of hand, foot and mouth disease (HFMD) in China. Monthly incidence HFMD cases in China from May 2008 to August 2018 were analysed with the SARIMA model. A seasonal variation of HFMD incidence was found from May 2008 to August 2018 in China, with a predominant peak from April to July and a trough from January to March. In addition, the annual peak occurred periodically with a large annual peak followed by a relatively small annual peak. A SARIMA model of SARIMA (1, 1, 2) (0, 1, 1)_12_ was identified, and the mean error rate and determination coefficient were 16.86% and 94.27%, respectively. There was an annual periodicity and seasonal variation of HFMD incidence in China, which could be predicted well by a SARIMA (1, 1, 2) (0, 1, 1)_12_ model.

Over the last decade, many outbreaks of hand, foot and mouth disease (HFMD) have been reported in countries of the Western Pacific Region, including Japan, Malaysia and Singapore, and across China (http://www.wpro.who.int/emerging_diseases/HFMD/en/, accessed on 12/8/2018). In China, HFMD has been categorised to class C notifiable diseases since May 2008, and the incidence ranks first among all of the notifiable diseases in China (http://www.nhfpc.gov.cn/zwgk/rdts/ejlist.shtml, accessed on 12/8/2018). This has prompted concerns that, without intervention, the public health impact and spread of HFMD will continue to intensify. Hence, given the high prevalence and morbidity, HFMD constitutes a substantial component of the burden of disease among children in China. Analysis of the long-term and seasonal variation of HFMD incidence is critical to identify the emerging concerns and provide evidence for prevention and control strategies on HFMD. The seasonal autoregressive-integrated moving average (SARIMA) model is widely used to model and predict the incidence of infectious diseases [1–3] including HFMD that has been explored in local areas of China [4–5]. However, the SARIMA to model and forecast monthly HFMD cases across China has not been reported. Therefore, in this study, we adopted the SARIMA model to model and forecast incidence of HFMD in China.

The monthly incidence data of HFMD from May 2008 are released by the National Health and Family Planning Commission of the People's Republic of China (http://www.nhfpc.gov.cn/zwgk/rdts/ejlist.shtml, accessed on 12/8/2018). In China, all HFMD cases verified by the clinical diagnosis must be reported within 24 h via the Infectious Disease Information Management System. Duplicate cards from the same person must be checked and deleted by the end of each month. Ethical approval is not required for this study because these are secondary data for public access.

The basic structure of a SARIMA model represents as SARIMA (*p*, *d*, *q*) (*P*, *D*, *Q*)_*S*_, where *p*, *d* and *q* are the autoregressive order, number of difference and moving average order, respectively; *P*, *D* and *Q* are the seasonal autoregressive order, number of seasonal difference and seasonal moving average order, respectively; and *S* is the length of the seasonal period. SARIMA was modelled with the Box and Jenkins strategy [6] including the following four stages. First, the Augmented Dickey–Fuller (ADF) method was used to determine whether the sequence was stationary, and logarithmic transformation and/or differencing could be adopted if the sequence was not stationary. In addition, the stationary sequence should not be a white noise. Ljung–Box portmanteau (or *Q*) test was used regarding the white noise tests of the original sequence and the residual series. The null hypothesis of the test is that the autocorrelation functions of the series have no significant elements for lags one through that specified by the lags option. Second, the autocorrelation coefficient (ACF) and partial autocorrelation coefficient (PACF) of the above stationary sequence were employed to identify the optional model parameters (*p*, *d*, *q* and *P*, *D*, *Q*) to establish one or more alternative models. Third, goodness-of-fit tests of the Akaike information criterion (AIC) and Bayesian information criterion (BIC) were used to select the best SARIMA model from competing alternatives, which should comply with the parametric test and the Ljung–Box portmanteau test that its residual series should be a white noise. Finally, mean error rate (MER) and determination coefficient (*R*2) were used to evaluate the accuracy of the most preferred model [1].

The seasonal pattern showed that the peak was mainly observed during April to July while the trough was mainly observed during January to March, and the annual peak occurred periodically with a large annual peak followed by a relatively small annual peak (Fig. 1). In addition, the ADF test showed that the time sequence was also not stationary, indicating that a long-term trend and seasonal pattern were present. After first-order regular difference and one seasonal difference, the time sequence was stationary (ADF test: *t* = −9.30, *P* < 0.01) (Supplementary Figure S1). In addition, the stationary sequence was not a white noise (*P* < 0.05) (Supplementary Figure S1) while the residual series was a white noise (*P* > < 0.05) (Supplementary Figure S2). Parameters of the ARIMA model were then identified with the ACF and PACF graphs (Supplementary Figure S1), and several candidate models were identified accordingly (Supplementary Table S1). Finally, SARIMA (1, 1, 2) (0, 1, 1)_12_ was identified as the most preferred model, with which the minimum values of AIC (25.20) and BIC (38.66) were found. In addition, all *P* values from parametric tests with SARIMA (1, 1, 2) (0, 1, 1)_12_ model were <0.05, and the residual is a white noise (all *P* values > 0.05).

**Fig. 1.**
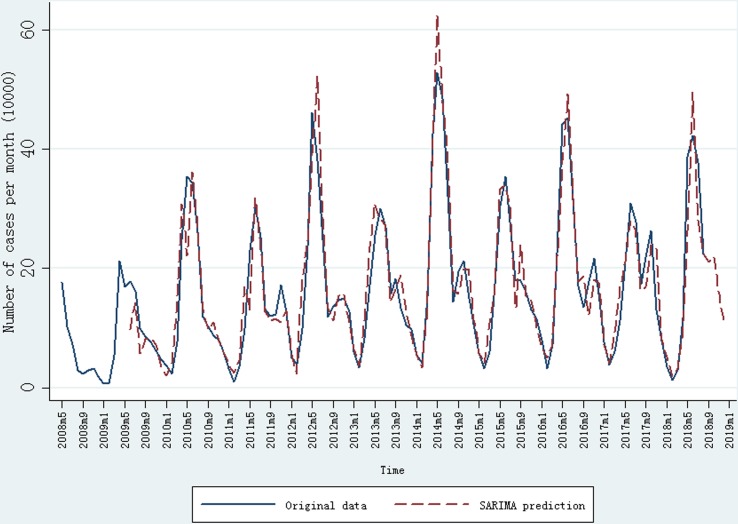
Monthly incidence of HFMD (May 2008 to August 2018) in China and prediction result of SARIMA model.

The SARIMA (1, 1, 2) (0, 1, 1)_12_ model was then adopted to forecast the incidence cases of HFMD in China. After first-order regular difference and one seasonal difference, 13 samples were lost in the SARIMA model construction. The observed incidence cases of HFMD and the forecasting results from SARIMA (1, 1, 2) (0, 1, 1)_12_ model are shown in Figure 1. The MER and determination coefficient were 16.86% and 94.27%, respectively. The predicted number of incidence HFMD cases from September 2018 to December 2018 was 211 711, 219 551, 156 981 and 106 303, respectively.

To our knowledge, this is the first study to model and forecast the monthly HFMD incidence cases with an ARIMA model across China from May 2008 to August 2018. A seasonal variation of HFMD incidence was found with a predominant peak between April and July. The annual peak occurred periodically with a large annual peak followed by a relatively small annual peak. The annual periodicity and seasonal variation could be modelled well with a SARIMA (1, 1, 2) (0, 1, 1)_12_ model.

Based on the monthly incidence data from January 2009 to December 2015 in Wuhan City, China, Peng *et al*. found that a SARIMA (1, 0, 1) (0, 1, 1)_12_ model adequately captured the pattern in the data and exhibited two peaks of activity over the forecast interval, including a major peak during April to June, and again a light peak for September to November [4]. HFMD monthly incidence data from January 2010 to June 2014 in Sichuan Province, China, showed that the SARIMA (1, 0, 1) × (0, 1, 0)_12_ model could be applied to forecast the HMFD incidence trend [5]. In this study, a SARIMA (1, 1, 2) (0, 1, 1)_12_ model was identified across China. The results suggested that the best-fit model for the whole country and for local areas may be different, which might arise from the temperature variation across China [7].

In China, overall 90% of the HFMD cases were children <5 years of age, and two serotypes Enterovirus A71 (EV-A71) and Coxsackievirus A16 (CV-A16) are responsible for the majority of these cases [8]. Two reasons may partially explain the finding that a large annual peak was followed by a relatively small annual peak. First, the vast majority of HFMD patients will have protective antibodies against the enterovirus serotype they have been infected with, thus the risk of repeated disease due to infection with the same enterovirus serotype is lower (http://www.chinacdc.cn/jkzt/crb/bl/szkb/zstd/201803/t20180326_159976.html, accessed on 12/6/218). Second, based on weekly reports of HFMD incidence from 31 provinces in Mainland China from 1 January 2009 to 31 December 2013, the multi-serotype time-series susceptible–infected–recovered epidemic models showed that the duration and strength of cross-protection following infection with EV-A71 or CV-A16 was estimated to be 9.95 weeks (95% confidence interval (CI) 3.31–23.40) in 68% of the population (95% CI 37–96%) [9]. In addition, for HFMD specifically, co-infection of a single individual with both serotypes is rarer than expected by chance, suggesting the existence of at least short-term cross-protection, and neutralisation assays have shown partial cross-reactivity between the EV-A71 and CV-A16 serotypes [9].

The strengths of this study included that we included nationwide HFMD data from 2008 to 2018, and validity of these data was supported by the mandatory notification system in China. However, there are also several limitations. First, the data included all cases verified by clinical or laboratory diagnosis, and it may miss infected individuals that have no access to healthcare professionals leading to under-reporting. Second, detailed information for HFMD cases are missing, such as age and sex, which preclude further analysis in this study. Third, data on the serotypes of enteroviruses like EV-A71 and CA-V16 are not available to us, thus sub-analysis of incidence trends by serotypes of enteroviruses is not conducted in this analysis.

China's self-developed inactivated monovalent EV-A71 vaccine was recently approved and is commercially available (http://www.nhfpc.gov.cn/qjjys/s3594r/201512/fa403581683d4b619bcee477aa15423e.shtml, accessed on 12/6/2018), and this is the first vaccine against EV-A71 in the world. The inactivated monovalent EV-A71 vaccine showed high efficacy (94.8–97.4%) against EV-A71-associated HFMD [8]. In addition, compared with no vaccination, routine paediatric EV-A71 vaccination would be very cost-effective in China [10]. Monovalent CV-A16 vaccine and bivalent EV-A71 and CV-A16 vaccine are under development. Therefore, with the inclusion of major circulating viruses in the development of multivalent HFMD vaccines, an increase in the success in HFMD control is anticipated.

In conclusion, there was an annual periodicity and seasonal variation of HFMD incidence in China, which could be predicted by a SARIMA (1, 1, 2) (0, 1, 1)_12_ model.

## Supplementary material

For supplementary material accompanying this paper visit https://doi.org/10.1017/S095026881800362X. click here to view supplementary material
