# Supplementary material for: Time-series modelling and forecasting of hand, foot and mouth disease cases in China from 2008 to 2018
Source: Epidemiol Infect. 2019 Jan 31;147:e82. doi: 10.1017/S095026881800362X (PMC6518604; doi:10.1017/S095026881800362X)
Supplement: Supplementary file 1 [file S095026881800362Xsup001.doc]

*Epidemiology and Infection,* **Time series modeling and forecasting of hand, foot, and mouth disease cases in China from 2008 to 2018,** C.W. Tian, H. Wang, W.M. Wang, X.M. Luo

Supplementary Material

**Supplementary Table 1** Comparison of candidate models

| Model | AIC | BIC | Parametric test | | Residual series are white noise at lags(30) |
| --- | --- | --- | --- | --- | --- |
|  |  |  | Parameter | P value |  |
| sarima(2,1,2) (1,1,1)_12_ | 28.60 | 47.44 | AR1  AR2  MA1  MA2  SAR1  SMA1 | <0.01  0.61  0.02  0.06  0.55  <0.01 | Yes |
| sarima(2,1,1) (1,1,1)_12_ | 29.57 | 45.72 | AR1  AR2  MA1  SAR1  SMA1 | <0.01  <0.01  <0.01  0.34  <0.01 | Yes |
| sarima(2,1,0) (1,1,1)_12_ | 33.50 | 46.96 | AR1  AR2  SAR1  SMA1 | <0.01  <0.01  0.73  <0.01 | Yes |
| sarima(2,1,2) (0,1,1)_12_ | 26.89 | 43.04 | AR1  AR2  MA1  MA2  SMA1 | <0.01  0.63  0.02  0.05  <0.01 | Yes |
| sarima(2,1,2) (1,1,0)_12_ | 30.10 | 46.25 | AR1  AR2  MA1  MA2  SAR1 | 0.01  0.64  0.03  0.09  <0.01 | Yes |
| sarima(2,1,1) (0,1,1)_12_ | 28.34 | 41.80 | AR1  AR2  MA1  SMA1 | <0.01  <0.01  <0.01  <0.01 | Yes |
| sarima(2,1,1) (1,1,0)_12_ | 31.13 | 44.59 | AR1  AR2  MA1  SAR1 | <0.01  <0.01  <0.01  <0.01 | Yes |
| sarima(2,1,0) (0,1,1)_12_ | 31.61 | 42.38 | AR1  AR2  SMA1 | <0.01  <0.01  <0.01 | Yes |
| sarima(2,1,0) (1,1,0)_12_ | 36.93 | 47.69 | AR1  AR2  SAR1 | <0.01  <0.01  <0.01 | Yes |
| sarima(1,1,2) (1,1,1)_12_ | 26.95 | 43.10 | AR1  MA1  MA2  SAR1  SMA1 | <0.01  <0.01  <0.01  0.58  <0.01 | Yes |
| sarima(1,1,1) (1,1,1)_12_ | 38.43 | 51.88 | AR1  MA1  SAR1  SMA1 | 0.26  <0.01  0.81  <0.01 | No |
| sarima(1,1,0) (1,1,1)_12_ | 43.64 | 54.40 | AR1  SAR1  SMA1 | 0.03  0.54  <0.01 | No |
| sarima(1,1,2) (0,1,1)_12_ | 25.20 | 38.66 | AR1  MA1  MA2  SMA1 | <0.01  <0.01  <0.01  <0.01 | Yes |
| sarima(1,1,2) (1,1,0)_12_ | 28.44 | 41.89 | AR1  MA1  MA2  SAR1 | <0.01  <0.01  <0.01  <0.01 | Yes |
| sarima(1,1,1) (0,1,1)_12_ | 42.39 | 53.15 | AR1  MA1  SMA1 | <0.01  <0.01  <0.01 | No |
| sarima(1,1,1) (1,1,0)_12_ | 42.93 | 53.69 | AR1  MA1  SAR1 | 0.21  <0.01  <0.01 | No |
| sarima(1,1,0) (0,1,1)_12_ | 41.95 | 50.02 | AR1  SMA1 | 0.02  <0.01 | No |
| sarima(1,1,0) (1,1,0)_12_ | 47.97 | 56.05 | AR1  SAR1 | 0.06  <0.01 | No |
| sarima(0,1,2) (1,1,1)_12_ | 35.44 | 48.90 | MA1  MA2  SAR1  SMA1 | 0.02  <0.01  0.68  <0.01 | Yes |
| sarima(0,1,1) (1,1,1)_12_ | 38.41 | 49.18 | MA1  SAR1  SMA1 | <0.01  0.82  <0.01 | No |
| sarima(0,1,0) (1,1,1)_12_ | 46.37 | 54.45 | SAR1  SMA1 | 0.38  <0.01 | No |
| sarima(0,1,2) (0,1,1)_12_ | 33.60 | 44.37 | MA1  MA2  SMA1 | 0.01  <0.01  <0.01 | Yes |
| sarima(0,1,2) (1,1,0)_12_ | 38.02 | 48.79 | MA1  MA2  SAR1 | 0.25  <0.01  <0.01 | Yes |
| sarima(0,1,1) (0,1,1)_12_ | 36.45 | 44.53 | MA1  SMA1 | <0.01  <0.01 | No |
| sarima(0,1,1) (1,1,0)_12_ | 43.40 | 51.48 | MA1  SAR1 | <0.01  <0.01 | No |
| sarima(0,1,0) (0,1,1)_12_ | 45.03 | 50.42 | SMA1 | <0.01 | No |
| sarima(0,1,0) (1,1,0)_12_ | 49.64 | 55.02 | SAR1 | <0.01 | No |

**
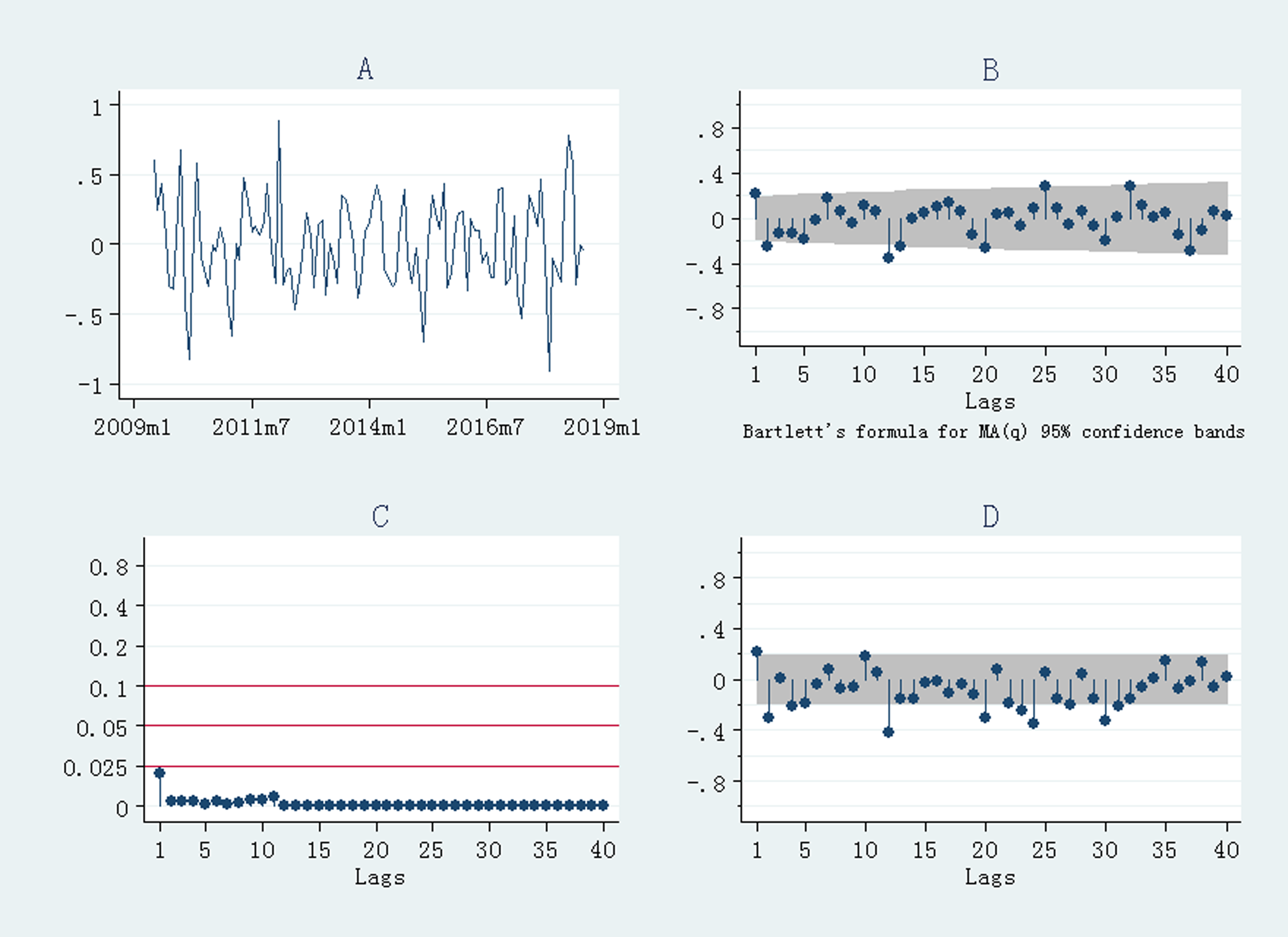
**

**Supplementary Figure 1** Diagnostics for HFMD after first-order regular difference and one seasonal difference. (A) stationary test; (B) autocorrelation coefficient graph; (C) white noise test; (D) partial autocorrelation coefficient graph.


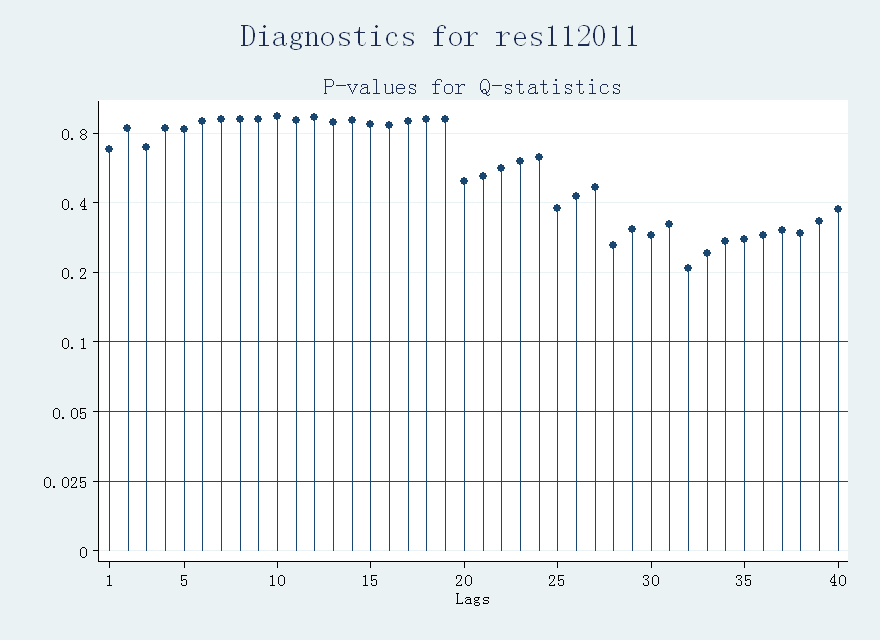


**Supplementary Figure 2** White noise tests of residual series (res112011).
